# Changes in peripheral immune populations during pregnancy and modulation by probiotics and ω-3 fatty acids

**DOI:** 10.1038/s41598-020-75312-1

**Published:** 2020-10-30

**Authors:** A. Forsberg, T. R. Abrahamsson, L. Nilsson, J. Ernerudh, K. Duchén, M. C. Jenmalm

**Affiliations:** 1 Division of Neuro and Inflammation Sciences, Department of Clinical and Experimental Medicine, Linköping University, Linköping, Sweden; 2 Department of Paediatrics, Department of Clinical and Experimental Medicine, Linköping University, Linköping, Sweden; 3 Department of Clinical and Experimental Medicine, Allergy Centre, Linköping University, Linköping, Sweden; 4 Department of Clinical Immunology and Transfusion Medicine, Department of Clinical and Experimental Medicine, Linköping University, Linköping, Sweden

**Keywords:** Immunology, Microbiology, Diseases, Medical research

## Abstract

Allergic diseases have become a major health problem, partly due to reduced microbial stimulation and a decreased dietary ω-3/ω-6 long-chain polyunsaturated fatty acid ratio. Prenatal exposures have been reported to influence allergy development, possibly induced via changes in maternal immune regulation. In a randomized double-blind placebo-controlled multicenter allergy prevention trial (PROOM-3), pregnant women were recruited at gestational week 20, and randomized to four study groups, one receiving both *L. reuteri* oil drops and ω-3 PUFA capsules (n = 22), the second receiving ω-3 PUFA supplementation and placebo regarding *L. reuteri* (n = 21), the third receiving *L. reuteri* and placebo regarding ω-3 PUFA (n = 22) and the fourth group receiving placebo capsules and placebo oil drops (n = 23). In this substudy, supplemental and pregnancy-related effects on maternal peripheral immune cell populations during pregnancy were assessed by flow cytometry immune phenotyping at gestational week 20, 32 and 4 days after delivery. The numbers of activated and regulatory T (Treg) cells (CD45RA^−^ Foxp3^++^/CD45RA^+^Foxp3^+^) were reduced after delivery, with the lowest count in the *L. reuteri* supplemented group compared with the placebo group 4 days after delivery, while the ω-3 PUFA group did not differ from the placebo group. Several treatment-independent changes were observed during and after pregnancy in lymphocytes (CD4^+^/8^+^/19^+^/56^+^/45RA^+/−^), CD14^+^16^+/−^ monocytes, and in subpopulations of T helper cells (Th) CD4^+^CD45RA^−^Tbet^+^ (Th1) and CD4^+^CD45RA^−^RORC^+^ (Th17) cells. In conclusion, probiotic supplementation to the mother during the second half of pregnancy resulted in immunomodulatory effects among activated and resting Treg cells. Furthermore, several systemic immune modifying effects of pregnancy were observed.

## Introduction

Allergic diseases have become an important health issue in affluent parts of the world^1^. In individual predisposition, genetic factors play an important role, however changes in the genotype alone cannot explain such a prompt escalation in the allergy prevalence. Thus, loss of protective features or presence of new risk factors must cause the increasing prevalence of these diseases since the middle of the last century^2–6^. A reduced amount and diversity of microbial encounters and a decreased dietary ω-3-/ω-6 long-chain polyunsaturated fatty acid ratio could be one of the key factors in the development of a deviated immune maturation^7,8^. Based on these hypotheses, both probiotics and ω-3 fatty acids have been used in infant allergy prevention trials^9–13^. According to the DOHaD theory, i.e. the developmental origins of health and disease, this can impact the developing fetus since both genetic and prenatal environmental factors may interact to influence disease development^14^.

Pre- and postnatal supplementation with probiotics have shown promising preventive effects on infant eczema development in several independent studies, as reviewed in^15,16^ Several allergy prevention studies^9–11^ also indicate that ω-3 fatty acid supplementation may have a protective effect on the development of allergic disease, as reviewed in^12,13^, although the results are inconsistent^17^. Particularly, supplementation during the pregnancy period seems to be of essential importance for the allergy preventive effects of probiotics and ω-3 fatty acids^2,3,7^, possibly due to the close immunological contact between the mother and the fetus^18,19^. In addition, probiotics and ω-3 fatty acid supplementation may modify the gut microbiota and thereby influence immune development^20,21^. However, the effects of probiotic supplementation on the gut microbiota diversity and composition have not been fully elucidated yet^15,22^. Furthermore, probiotics may signal via TLR2 on epithelial cells to decrease gut permeability and on intestinal DCs to induce Treg activity^2^. Also, several immunomodulatory mechanisms of ω-3 fatty acids have been described, including decreased inflammation, possibly mediated through signalling via GPR120 on macrophages, reviewed in^2^. In addition, resolution of inflammation may be due to actions of resolvins, protectins, and maresins, which are suggested to block neutrophil recruitment, promote infiltration and activation of monocytes, and induce phagocytosis and lymphatic clearance of apoptotic neutrophils by activated macrophages^23^, inhibition of Th2 activation and promotion of Th1 and Treg activity^2^.

Furthermore, immune mediators, secreted systemically and locally during pregnancy, may affect the immunological environment at the fetal-maternal interface, thereby influencing the developing fetal immune system^24^. Pregnancy is a unique situation in which the mother and the semi-allogeneic fetus peacefully coexist. Numerous mechanisms are needed, including fetal, maternal and placental, to protect the fetus from immunological recognition, rejection and infection^18,19^. Cells from the local environment, such as uterine/decidual T cells^25^, macrophages^26,27^, and natural killer (NK) cells^28,29^, contribute to modulating the environment at the fetal maternal interface to sustain a successful pregnancy. In addition to mediating hormonal, nutritional and oxygen support of the fetus, the placenta also plays an important immunomodulatory role^30^. The local immune environment is also reflected in peripheral blood, from which many immune cells are recruited to sustain local immune tolerance^30^. Moreover, as clinical symptoms of immune mediated diseases, such as rheumatoid arthritis, psoriasis and multiple sclerosis, are reduced during pregnancy, the effect of pregnancy on the maternal immunity is not limited to the maternal–fetal interface^31–33^. More detailed studies on how systemic immunity is modulated during pregnancy are required since existing studies show conflicting results including monocytes, NK-cells and T- helper cell subsets regarding changes in peripheral immune cell populations^30,34–36^.

The allergy preventive effects of prenatal probiotic and ω-3 fatty acid supplementation are likely induced via changes in maternal immune regulation^7,24^. However, most previous intervention studies started prenatal probiotic supplementation during the last trimester of pregnancy^15^ although one study started as early as in gestational week 14–16^21^. If prenatal microbial exposure is vital for the preventive effect, starting supplementation already from the second trimester of pregnancy, when circulating fetal T cells have developed, may have a more powerful allergy preventive effect^15,24^. Furthermore, probiotic and ω-3 polyunsaturated fatty acid administration during pregnancy may act synergistically via immunoregulatory and anti-inflammatory mechanisms, respectively^7^.

The aims of the study were to investigate how maternal peripheral immunity is affected by pregnancy, and by probiotic and ω-3 fatty acid supplementation. We have used flow cytometry and a broad panel of immune markers to map peripheral immune cell populations during the course of pregnancy in women participating in a randomized double-blind placebo-controlled multicenter allergy prevention trial involving supplementation with the probiotic *L. reuteri* and ω-3 fatty acid from gestational week 20.

## Material and methods

### Participants in the study

The mothers participated in a prospective randomized double-blind placebo-controlled multicenter allergy prevention trial (PROOM-3), conducted at the Department of Pediatrics and Allergy Centre at the University Hospital in Linköping and in the county hospitals of Motala, Jönköping and Norrköping in Sweden. The clinical evaluation of allergy in the children is still ongoing. Families with at least one parent or sibling with clinical symptoms or history of allergic disease was invited to participate in the study. The mothers in this sub study were recruited from 2011 to 2018. The pregnant women were included in the study at gestational week 20. The women were randomized to four study groups, one receiving both *L. reuteri* oil drops and ω-3 PUFA capsules, the second receiving ω-3 PUFA supplementation and placebo regarding *L. reuteri*, the third receiving *L. reuteri* and placebo regarding ω-3 PUFA, and the fourth group receiving placebo capsules and placebo oil drops. The *L. reuteri* or placebo oil drops were given to the mothers during pregnancy, from gw 20 to delivery, as well as the ω-3 or placebo capsules were given to the mothers during pregnancy. The ω-3 PUFA treatment comprised of maternal supplementation of three capsules of Pikasol (1 g capsules containing 640 mg ω-3 PUFA) twice daily during pregnancy and lactation, while the placebo capsules contained similar amounts of olive oil. The *L. reuteri* supplementation comprised of 10^9^ colony forming units (CFU) *L. reuteri* DSM 17938 (corresponding to 20 droplets × 2 daily) suspended in oil (refined coconut and peanut oil) to the mothers during pregnancy and 10^8^ CFU (5 drop daily) to the children during the first years of life. The placebo drops contained similar amounts of oil without *L. reuteri.* An independent statistician not otherwise involved in the study using blocked randomization with block sizes of eight prepared the computer-generated randomization list. The same statistician keeps the randomization list. Thus, the study was blinded to investigators, study nurses and all clinical staff. The power of the study was calculated using two previous intervention studies^9,37^. The primary aim of this sub study is to assess the effect of a combined maternal supplementation with two supplements, which by their own have previously shown to prevent the development of IgE associated disease in childhood, on the maternal peripheral immune cell population. The participants in this sub study of immune cell populations were recruited in Linköping and Motala and are described in Table 1. The clinical characteristics did not differ significantly between the supplemented groups (*p* > 0.05). Sample size was determined with the help from an independent statistician and based on results from two previous immune studies^38,39^. No adverse events were reported during study period. Blood samples were collected from the pregnant women at gestational week 20, 32 and postpartum (4 days after delivery). Only mothers with two or more fresh whole blood samples were included in the sub study, Table 1. Non-pregnant age-matched women (n = 22), not taking hormonal contraceptives were included as control subjects, for clinical characteristics see Supplementary Table I.

**Table 1:** Characteristics of the study participants.

	Ω3 + *L. Reuteri* n = 22	Ω3 + Placebo n = 21	Placebo + Placebo n = 22	Placebo + *L. Reuteri* n = 23
Partus at week (mean)	40	39	39	39
Mothers age at inclusion (years/mean)	29	30	29	29
No of weeks with study product (mean)	19.6	18.6	18.5	18.8
Birth weight (kg/mean)	3.40	3.30	3.37	3.25
Birth length (cm/mean)	49.5	49.5	49.0	49.5
Caesarean (n/%)	1 (5%)	3 (14%)	3 (14%)	2 (9%)
First born (n/%)	12 (55%)	17 (81%)	13 (59%)	11 (47%)
Animals in household (n/%)	3 (14%)	7 (33%)	3 (27%)	8 (35%)
Smoking parent (n)	0	0	0	0
Maternal allergic disease (n/%)	16 (72%)	12 (57%)	13 (59%)	11 (47%)

*No significant differences between groups using Kruskal–Wallis.

### Ethical aspects

Written informed consent was obtained from both parents at the inclusion of the study. The study was approved by the Regional Ethics Committee for Human Research in Linköping (Dnr 2011/45–31) and registered at ClinicalTrials.gov (Identifier: NCT01542970, 02/03/2012). All experiments were performed in accordance with the Helsinki Declaration ethical principles for medical research.

### Flow cytometry

Flow cytometry was used to analyze immune cell populations in peripheral blood. Whole blood was incubated for 30 min at 4 °C with surface antibodies (details regarding the antibodies are given in Supplementary Table II). NH_4_Cl was added and incubated for 15 min at RT to lyse erythrocytes. Cells were centrifuged for 5 min at 500 g and 7 °C and washed in PBS with 5% FCS. Secondary antibodies were added when appropriate and incubated for 30 min at 4 °C. After washing as described above, cells were fixed (Foxp3 Staining Buffer Set, eBiosciences, San Diego, CA, USA) for 30 min at 4 °C. After a centrifugation step, a second centrifugation with permeabilizing solution (Foxp3 Staining Buffer Set, eBiosciences) was performed. Antibodies against intracellular antigens were then added for 30 min, 4 °C. After washing and centrifugation cells were resuspended in PBS with 5% FCS and analyzed. For the Trucount tubes (BD, Franklin Lakes, New Jersey, USA), the antibodies were placed in 50 µl blood and incubated for 30 min at 4 °C. 450 µl lysing solution (BD FACS Lysing Solution, BD) was used to lyze red blood cells and then samples were analyzed.

Within the lymphocyte population (based on forward (FSC) and side scatter (SSC) characteristics), the proportion and total number of major populations were defined in TrueCount tubes as follows (clones within brackets); B cells, APC-conjugated anti-CD19 (clone SJ25C1); NK cells, PE-conjugated anti-CD56 (clone B159, NK cells were also CD3-); T cells, APCCy7-conjugated anti-CD3 (SK7); T cytotoxic cells, FITC-conjugated anti-CD8 (clone SK1); T helper cells, PECy7-conjugated anti-CD4 (SK3). CD4 and CD8 populations were defined by co-expression of CD3. All these antibodies were from Beckton Dickinson, Franklin Lakes, New Jersey, USA. Within the CD3^+^CD4^+^ population, the naive and memory cell population was determined using v450-conjugated anti-CD45RA (HI100) (Beckton Dickinson). Expression of the intracellularly expressed T helper cell lineage markers were stained using PE-conjugated anti-GATA3 (TWAJ), eFlour 660-conjugated anti-Tbet (eBio4B10), PE-conjugated anti-RORC (AFKJS-9), FITC-conjugated anti-Foxp3 (PCH101) (all from eBiosciences) was in the CD45RA^+^ naïve (undifferentiated) and CD45RA^−^ memory (differentiated) CD3^+^CD4^+^ T helper cell population. The percentage of T-bet, GATA-3 and RORC expressing populations was determined using the CD3^+^CD4^+^CD45RA^+^ population as a population without cells expressing the markers (naïve cell population) and comparing these with CD3^+^CD4^+^CD45RA^−^ memory population where differentiated cells reside. T regulatory cells (Tregs) were defined as CD4^dim^CD25^hi^Foxp3^+^^40^ and subtypes of Tregs were also subdivided into CD3^+^CD4^+^CD45RA^+/−^Foxp3^+/++^, i.e. resting and activated Tregs^41^, respectively. For gating strategies, see Supplementary Fig. 1. Monocytes were determined based on expression of surface antigens detected using FITC conjugated anti-CD14 (M0P9, BD) and PerCpCy5.5 conjugated anti-CD16 (3G8, BD). Monocytes were gated first on FSC and SSC and CD14^+^ expression, and subtypes of monocytes were subdivided based on CD14^+^CD16^+/−^ expression. All antibodies used in the project are listed in Supplementary Table II. Data was acquired on a BD FACS CANTO II and analyzed using Kaluzaa 1.2 (Beckman Coulter).

**Figure 1:**
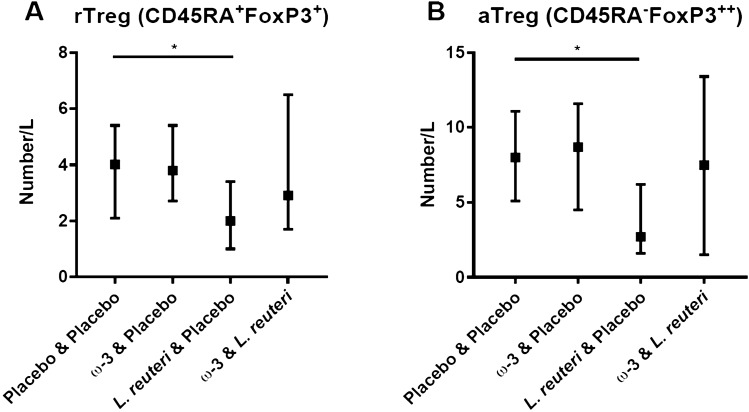
(**A**) Resting Tregs (CD4^+^CD45RA^+^Foxp3^+^) (**B**) Activated Tregs (CD4^+^CD45RA^−^Foxp3^++^) after delivery in the four treatment groups (approximately 20 weeks of supplementation). Median values and interquartile ranges are shown. The Kruskal–Wallis test was used for comparison between treatment groups. If the Kruskal–Wallis test was significant, differences between the placebo + placebo group and the other treatment groups were analyzed with Mann–Whitney *U* test and Bonferroni correction for multiple comparisons.

### Statistical analysis

An independent statistician performed the randomized statistical analyses comparing the treatment groups, since the study was blinded for the investigators during the actual study. All other statistical analyses were performed by A.F. Study groups were compared longitudinally during pregnancy using the Friedman test. The Kruskal–Wallis test was used for comparison between treatment groups. If the Kruskal–Wallis test was significant differences between the placebo + placebo group and other treatment groups were analyzed with Mann–Whitney U test and Bonferroni correction for multiple comparisons. Clinical variables were compared using one-way ANOVA and chi-square test, Table 1. Differences between pregnant and non-pregnant women were compared using the Mann–Whitney *U* test. *P* values ≤ 0.05 were considered statistically significant. Calculations were performed with a SPSS statistical package version 24.0: SPSS Inc, Chicago, Ill and GraphPad Prism 7 version 7.03, GraphPad Software, Inc La Jolla, CA, USA.

## Results

### Supplementation with *L. reuteri* from gestational week 20 alters activated Treg (CD4^+^*CD45RA*^*-*^*Foxp3*^++^) and resting Treg (CD4^+^*CD45RA*^+^*Foxp3*^++^) numbers in peripheral blood after delivery

Four days after delivery, a significant difference was noted for Treg cells when considering all groups including placebo and groups supplemented with ω-3 and probiotics during pregnancy (from week 20 to delivery). Comparing the placebo + placebo group with the treatment groups (Mann–Whitney *U* test and Bonferroni correction) at 4 days after delivery revealed that the *L. reuteri* group had significantly lower numbers of resting Treg cells (CD4^+^CD45RA^+^Foxp3^+^, adjusted *p* = 0.036, Fig. 1A) as well of activated Treg cells (CD4^+^CD45RA^−^Foxp3^+^, adjusted *p* = 0.021, Fig. 1B). In contrast, the ω-3 group did not differ compared with the placebo group. Besides Treg cells, other lymphocyte and monocyte cell populations were not affected by the supplementation (data not shown).

### *Systemic changes in subsets of lymphocytes (CD4*^+^*/8*^+^*/19*^+^*/56*^+^*) during pregnancy and compared to non-pregnant women*

To determine how the systemic immunity was affected by pregnancy, several cell types in peripheral blood were investigated longitudinally (Table 2 and Supplementary Fig. 2). The total amount of lymphocytes increased significantly from gw 20 to 4 days after delivery, but the proportions were significantly higher in non-pregnant than pregnant women (*p* < 0.001 for both comparisons, Table 2, Supplementary Fig. 2A,B). The percentage and total number of CD4^+^ T helper cells decreased from gw 20 to gw 32 but increased from gw 32 to 4 days after delivery (*p* < 0.001 for all time points, Table 2, Supplementary Fig. 2C,D), and the percentage was higher in pregnant than non-pregnant women, although the absolute numbers were lower in the pregnant than in non-pregnant women at gw 20. The frequencies of CD8^+^ cytotoxic T cells increased from gw 20 to 4 days after delivery (Table 2, Supplementary Fig. 2E,F). The total number of these cells were lower in pregnant than non-pregnant women (i.e. gw 20 and gw 32). The proportions and numbers of CD19^+^ B cells were increased during pregnancy; higher percentages were observed at gw 20 and gw 32 than in non-pregnant women (Table 2, Supplementary Fig. 2G,H). When investigating NK cells, the CD56^+^ proportions and numbers were lower during pregnancy than in non-pregnant women (*p* < 0.001 for all comparisons, Table 2, Supplementary Fig. 2I,J). Within the CD56^+^ population, the proportions of CD56^dim^ cells were lower in pregnant than non-pregnant women, with similar patterns for the absolute number of cells (*p* < 0.001 for all cases, Table 2, Supplementary Fig. 2K–L). In contrast, the frequencies and numbers of CD56^hi^ cells were similar in pregnant and non-pregnant women, although a slight increase in frequencies was observed from gw 20 to after delivery (Table 2, Supplementary Fig. 2M,N).

**Table 2:** Proportions and number of lymphocytes in peripheral blood during pregnancy and in non-pregnant women (median and interquartile range).

	w20 (% SEM)	w20 (n/L 10 × 6)	w32 (%)	w32 (n/L 10 × 6)	Partus (%)	Partus (n/L 10 × 6)	Non-pregnant (%)	Non-pregnant (n/L 10 × 6)
Lymphocytes (% of leukocytes and number)	21 (17–28)	1655 (1286–1934)	21 (16–26)	1652 (1356–1997)	26 (22–36)	1906 (1435–2175)	36 (28–39)	2012 (1624–2369)
CD3^+^ CD4^+^ (% of lymphocytes and number)	48 (43–52)	787 (585–959)	48 (43–51)	786 (580–963)	50 (44–56)	915 (670–1141)	44 (40–46)	843 (685–979)
CD3^+^ CD8^+^ (% of lymphocytes and number)	25 (21–29)	387 (305–497)	25 (21–29)	408 (313–488)	26 (22–30)	486 (358–607)	26 (20–28)	478 (412–575)
CD19^+^ (% of lymphocytes and number)	11 (8.7–12)	175 (119–220)	11 (8.4–13)	174 (123–217)	7.3 (6.0–9.3)	136 (108–168)	6.9 (5.8–8.3)	144 (111–188)
CD56^+^ (% of lymphocytes and number)	6.3 (4.5–8.7)	99 (7–145)	6.3 (4.7–8.5)	100 (74–138)	6.0 (4.4–7.9)	105 (74–149)	10 (6.9–14)	174 (126–285)
CD56dim (% of lymphocytes and number)	5.0 (3.6–7.2)	80 (60–120)	4.8 (3.7–6.7)	80 (60–120)	4.7 (3.6–6.2)	80 (60–120)	8.2 (5.7–11)	150 (110–220)
CD56bright (% of lymphocytes and number)	0.6 (0.4–0.9)	10 (7–14)	0.6 (0.4–0.9)	9 (6–14)	0.6 (0.4–0.9)	12 (7–17)	0.5 (0.4–0.8)	9 (7–14)
Memory Th (% of CD3^+^ CD4^+^ and number)	54 (45–64)	420 (283–519)	52 (42–61)	382 (304–469)	52 (41–61)	432 (345–568)	58 (52–64)	542 (385–620)
Naïve Th (% of CD3^+^ CD4^+^ and number)	46 (35–54)	351 (240–441)	47 (39–57)	366 (253–501)	49 (39–59)	453 (298–594)	41 (33–48)	353 (267–452)
Memory GATA3^+^ (% of CD3^+^ CD4^+^ and number)	1.6 (0.6–3.4)	6.1 (2.7–11)	1.2 (0.54–3.2)	5.6 (2.1–12)	1.4 (0.62–2.9)	7.3 (2.5–14)	2.5 (1.0–5.9)	9.8 (5.5–23)
Memory RORC^+^ (% of CD3^+^ CD4^+^ and number)	0.12 (0.07–0.25)	0.46 (0.25–0.95)	0.2 (0.11–0.42)	10.1 (4.9–29.2)	0.16 (0.1–0.3)	0.7 (0.4–1.3)	0.08 (0.06–0.15)	0.5 (0.3–0.6)
Memory Tbet^+^ (% of CD45RA- and number)	1.7 (0.85–3.0)	5.9 (2.9–11.8)	1.4 (0.7–3.0)	6.1 (2.52–11.8)	1.3 (0.6–2.6)	5.8 (2.2–11.9)	2.4 (1.1–5.4)	11.7 (5.3–21.7)
CD4^dim^CD25^hi^Foxp3^+^ (% of CD3^+^ CD4^+^ and number)	1.9 (1.4–2.4)	13.7 (9.3–18.2)	1.6 (1.2–2.2)	12.0 (9.0–18.0)	0.84 (0.6–1.2)	7.3 (5.2–10.2)	1.9 (1.6–2.7)	15.2 (11.8–22.9)
(CD45RA^+^ Foxp3^+^) + (CD45RA^−^ Foxp3^++^) (r^+^ aTreg) (% of CD3^+^ CD4^+^ and number)	0.9 (0.6–1.4)	6.4 (4.2–10.1)	0.5 (0.4–0.8)	4.0 (2.7–5.7)	1.2 (0.6–1.7)	10.9 (4.8–15.0)	2.0 (1.4–2.6)	16.0 (12.2–22.3)
aTreg (% of CD3^+^ CD4^+^ and number)	0.36 (0.17–0.59)	2.41 (1.32–4.06)	0.36 (0.024)	0.25 (0.1–0.4)	0.7 (0.4–1.1)	6.6 (2.3–10.4)	1.1 (0.8–1.7)	9.5 (6.2–14.4)
rTreg (% of CD3^+^ CD4^+^ and number)	0.65 (0.32–0.77)	4.0 (2.2–6.3)	0.24 (0.16–0.34)	1.8 (1.1–2.6)	0.4 (0.2–0.5)	3.1 (1.7–4.9)	0.7 (0.6–0.9)	5.6 (4.6–8.8)
CD14^+^ (of total)	6.6 (5.7–8.4)		6.0 (5.1–7.0)		5.5 (4.2–6.5)		11.6 (8.1–21)	
CD14^+^ CD16^−^ (% of CD14^+^)	93 (91–95)		93 (90–95)		95 (93–96)		93 (91–96)	
CD14^+^ CD16^+^ (% of CD14^+^)	6.1 (4.2–8.3)		5.8 (4.6–8.1)		4.9 (3.1–6.6)		5.2 (2.8–6.1)

**Figure 2:**
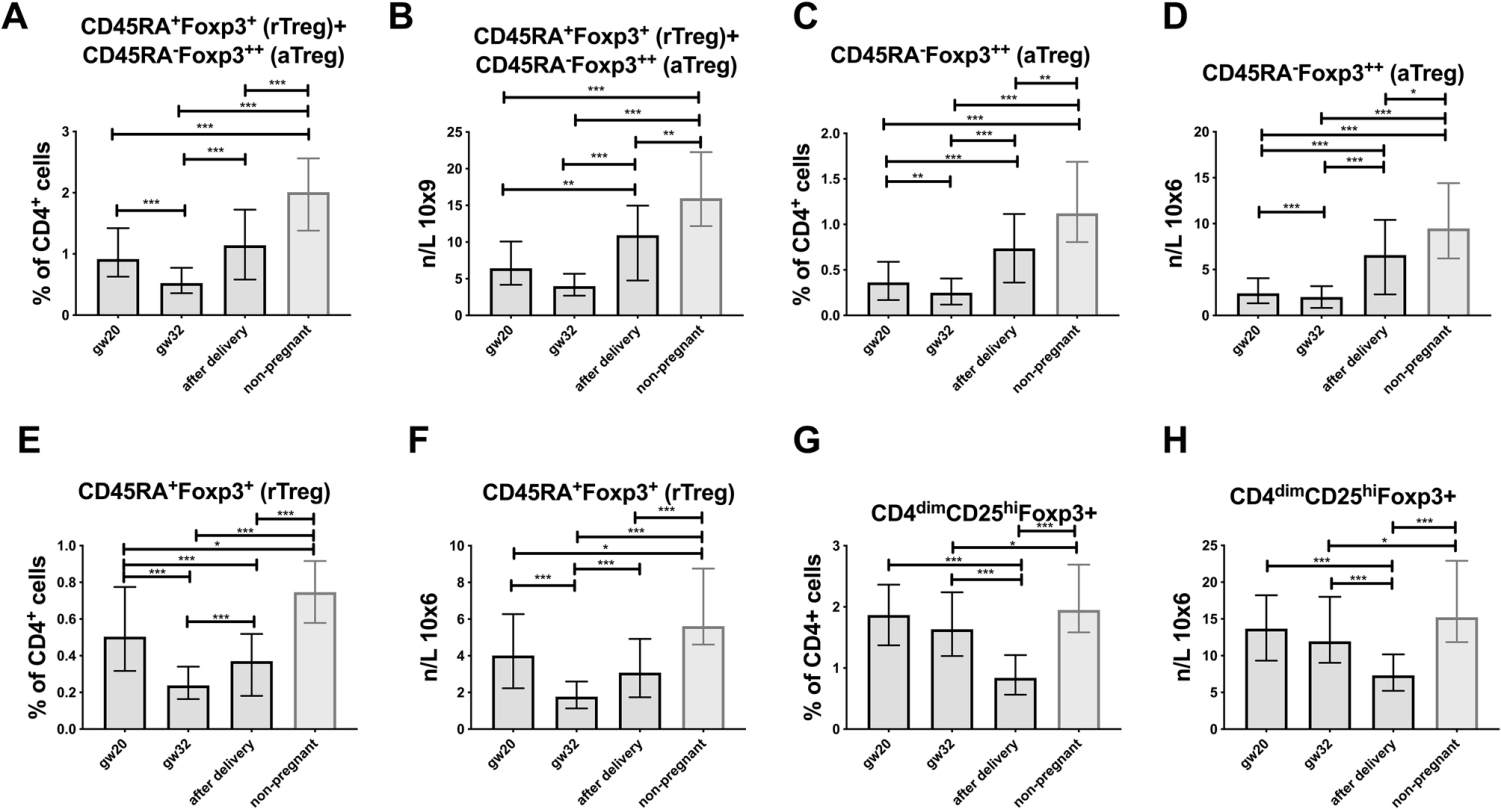
Different Treg populations during pregnancy and in non-pregnant women (**A**) percentage of aTreg (CD4^+^CD45RA^–^Foxp3^++^) and rTreg (CD4^+^CD45RA^+^Foxp3^+^) (**B**) number of aTreg (CD4^+^CD45RA^–^Foxp3^++^) and rTreg (CD4^+^CD45RA^+^Foxp3^+^) (**C**) percentage of aTreg (CD4^+^CD45RA-Foxp3^++^) (**D**) number of aTreg (CD4^+^CD45RA^−^Foxp3^++^) (**E**) percentage of rTreg (CD4^+^CD45RA^+^Foxp3^+^) (**F**) number of rTreg (CD4^+^CD45RA^+^Foxp3^+^) (**G**) percentage of (CD4^dim^CD25^hi^Foxp3^+^) (**H**) number of (CD4^dim^CD25^hi^Foxp3^+^). Median values and interquartile ranges are shown. Mann–Whitney *U* test and Wilcoxon test were used for statistical comparisons.

### *Naïve and memory T helper cells (CD3*^+^*CD4*^+^*CD45RA*^*−*^*/*^+^*) in peripheral blood in pregnant and non-pregnant women*

The memory T helper (CD3^+^CD4^+^CD45RA^−^) frequencies decreased from gw 20 to gw 32 and from gw 20 to 4 days after delivery (*p* < 0.001 for both comparisons), while the naïve T helper (CD3^+^CD4^+^CD45RA^+^) proportions and numbers increased from gw 20 to gw 32 and from gw 20 to 4 days after delivery (*p* < 0.001 for both comparisons, Table 2 and Supplementary Fig. 3). Non-pregnant women had significantly higher proportions and numbers of memory T helper cells, as compared with women at 4 days after delivery and at gw 32, respectively, while the proportions and numbers of naïve T helper cells were lower in non-pregnant women as compared with women at partus (*p* < 0.05, Table 2, Supplementary Fig. 3A–D). The percentages of naïve T helper cells were also lower in non-pregnant women as compared with pregnant women at gw 32 (Table 2, Supplementary Fig. 3C).

**Figure 3:**
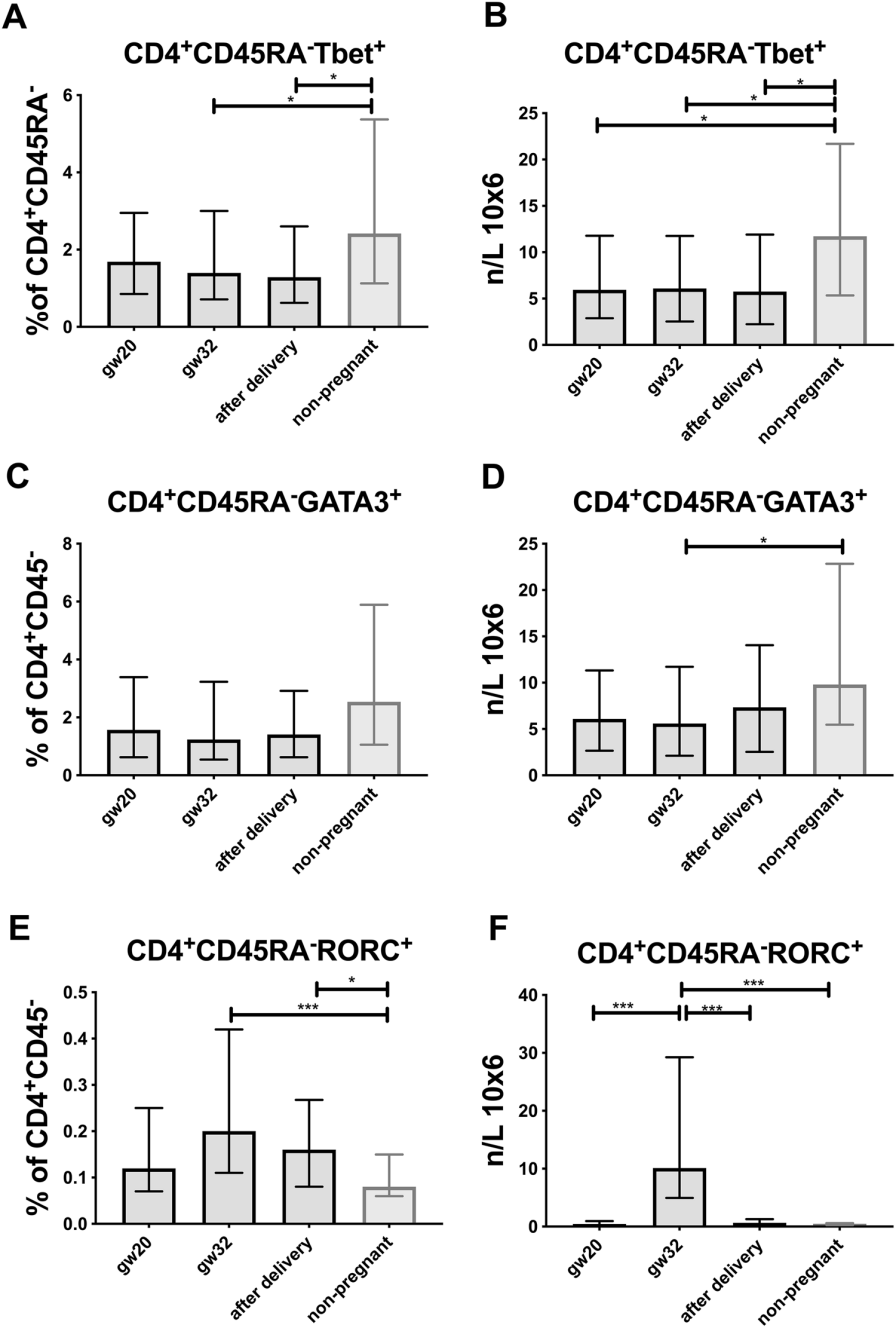
T helper cells during pregnancy and in non-pregnant women (**A**) percentage of memory Th1 cells (CD4^+^CD45RA^−^Tbet^+^) (**B**) number of memory Th1 cells (CD4^+^CD45RA^−^Tbet^+^) (**C**) percentage of memory Th2 cells (CD4^+^CD45RA^−^GATA3^+^) (**D**) number of memory Th2 cells (CD4^+^CD45RA^−^GATA3^+^) (**E**) percentage of memory Th17 cells (CD4^+^CD45RA^−^RORC^+^) (**F**) number of memory Th17 cells (CD4^+^CD45RA^−^RORC^+^). Median values and interquartile ranges are shown. Mann–Whitney *U* test and Wilcxon test were used for statistical comparisons.

### *Different populations of T regulatory cells (CD45RA*^*-*^* Foxp3*^++^*/CD45RA*^+^*Foxp3*^+^*) and (CD4*^*dim*^*CD25*^*hi*^*Foxp3*^+^*) are reduced during pregnancy in peripheral blood*

Several changes were observed among the different Treg populations during pregnancy and in comparison with non-pregnant women (Table 2). Both CD4^+^CD45RA^+^Foxp3^+^ (resting Tregs) and CD4^+^CD45RA^−^Foxp3^++^ (activated Tregs) were lowest in proportion and number at gw 32, significantly decreased from gw 20 and significantly higher 4 days after delivery, and when combining the two populations the same pattern was found (Fig. 2A–F, Table 2). In non-pregnant women, aTreg cells were significantly higher in percentage and number compared to pregnant women at all time points (Fig. 2C,D). The same pattern was observed for rTregs (Fig. 2E,F) and for the two populations, aTreg + rTreg (Fig. 2A,B).

The proportion and number of CD4^dim^CD25^hi^Foxp3^+^ cells were higher at gw 20 than at gw 32 and at 4 days after delivery. Non-pregnant control women had significantly higher percentage and total number of CD4^dim^CD25^hi^Foxp3^+^ cells than pregnant women in gw 32 and after delivery (Fig. 2G,H, Table 2).

### *Th1, Th2 and Th17 (CD4*^+^*CD45RA*^*-*^*Tbet*^+^*/GATA3*^+*/*^*RORC*^+*)*^* subpopulations during pregnancy and in comparison with non-pregnant women*

To investigate Th1/Th2/Th17 skewing during pregnancy, we determined the number and proportions of CD4^+^CD45RA^−^ memory cells expressing the corresponding lineage specific transcription factors Tbet, GATA3 and RORC (Table 2). The proportions of Tbet^+^ (Th1) and GATA3^+^ (Th2) CD4^+^CD45RA^−^ memory cells among CD4^+^CD45RA^−^ cells did not change significantly during pregnancy (Fig. 3A–D, Table 2). Similar patterns were observed for the absolute number of cells, except that the number of Th17-associated CD4^+^CD45RA^−^RORC^+^ cells were significantly increased at gw 32 compared to gw 20 and after delivery (Fig. 3E,F, Table 2). In addition, non-pregnant women had a significantly higher number of Tbet^+^ cells than pregnant women (Fig. 3A,B), and also a higher number of GATA3^+^ cells compared to pregnant women at gw 32 (Fig. 3C,D) conversely, the number of RORC^+^ cells were lower in non-pregnant women than at gw 32 and after delivery (Fig. 3E,F).

### *Changes in monocyte populations (CD14*^+^*/CD14*^+^*16*^+^*/*^*−*^*) during pregnancy*

The proportion of CD14^+^ monocytes among leukocytes decreased during pregnancy and was significantly lower in pregnant than non-pregnant women (Table 2). Within the CD14^+^ monocyte population, the CD14^+^16^−^ cell proportions were lower at gw 20 and gw 32 than at 4 days after delivery (*p* < 0.001). Conversely, the CD14^+^16^+^ cell proportions were higher at gw 20 and gw 32 compared to 4 days after delivery (*p* < 0.001). Non-pregnant women had a significantly lower percentage of this population of cells compared to pregnant women at gw 32 (Supplementary Fig. 4A–C).

## Discussion

In this study, we investigated how *L. reuteri* and ω-3 treatment affected peripheral immune cell populations during pregnancy as part of a randomized double-blind placebo controlled allergy prevention study. It has not previously been determined what effects probiotic and ω-3 treatment may have on peripheral immune cell populations in pregnant women. Previous research have highlighted the importance of prenatal supplementation for allergy preventive effects, and it has been suggested that these effects may be mediated via changes in maternal immune regulation^15^. Such immunomodulatory mechanisms have been poorly characterized, however.

In the present study we report that after around 20 weeks of supplementation during the second half of pregnancy, the numbers of activated and resting regulatory T cells in peripheral blood were lowest in the *L. reuteri* supplemented group. Additionally, treatment with ω-3 fatty acids should in theory be anti-inflammatory^2^ due to a competitive effect on the prostaglandin-signaling pathway (reviewed in^23,42,43^). However, we did not observe any effects of ω-3 fatty acid supplementation on peripheral blood immune cell populations during pregnancy. Possibly the ω-3 supplementation has other mechanisms of action than affecting cell number, frequencies or cell types studied and therefore not detected in this study.

Earlier studies have indicated that activated Tregs may have an increased migratory capacity, as shown by their chemokine receptor expression^44,45^. Almost all aTregs express the chemokine receptor CCR4 in contrast to the resting Tregs (CD45RA^+^Foxp3^+^)^44^. Other studies have shown that Treg cell express CCR7 and CCR9, which seem to be important in the thymic selection^46^ and in trafficking to the intestines^45,47^, respectively. Speculatively, the lower number of aTregs and rTregs in peripheral blood in the *L. reuteri* treated group could indicate that these cells have migrated from the periphery to other body compartments such as the fetal-maternal interface and the GI tract. The chemokine receptor expression of the Tregs would have been highly interesting to study, as well as their suppressive ability and cytokine expression, but these features were not included which is a limitation to our study. Dendritic cells in the lamina propria and mesenteric lymph nodes (MLNs) are known to contribute to the induction of Treg cells^48,49^ and their production of IL-10 and TGF-β under the influence of constitutive environmental signals^50^, which also include interaction with commensal bacteria^51^. In addition, probiotic supplementation have been proposed to have several immune modulatory effects such as Th1 and Treg promoting effects, diverting an unfavorable Th2 response, as reviewed in^15^. However, we were not able to detect any differences regarding supplementation between the other Th cell populations in this cohort.

Pregnancy is a unique immunological situation, as the semi-allogenic fetus must be tolerated by the maternal immune system^18^. Immune tolerance at the fetal-maternal interface is of essential importance and immune cells may be recruited from the periphery or induced locally to sustain tolerance. This implicates major changes in the maternal immune system, also reflected in systemic immunity^30^. In this study the different supplementation groups had similar longitudinal changes during pregnancy. The only exception was the differences among aTregs/rTregs after 20 weeks of probiotic supplementation. Thus, we monitored longitudinal changes among the combined treatment groups and made comparisons with non-pregnant control women, and were able to observe several changes in immune cell populations during pregnancy. In line with earlier observations, we noted an increase of the naïve cell subset (CD3^+^CD4^+^CD45RA^+^) and a decrease of memory subset (CD3^+^CD4^+^45RA^−^) among Th cells, suggesting a state of suppression of effector T cells with capacity to strongly respond to recall antigens^52^.

Our study investigated three different populations of Treg cells^41,53^. In humans, Treg cells are identified as CD4^+^ T helper cells expressing Foxp3 and high levels of the IL-2 receptor α-chain, CD25^54,55^. These cells are major immune regulators, suppressing many cell types including T cells^56^ via mechanisms that are incompletely understood but involving cell–cell contact through, for example, CTLA-4 as well as secretion of soluble mediators such as the cytokines interleukin-10 (IL-10), transforming growth factor-β (TGFβ), and IL-35^57^. Several strategies have been used to define T regulatory cells, as the key phenotypic markers CD25 and Foxp3 also are expressed upon activation of conventional CD4+ T cells, complicating the enumeration of ‘true’ Treg cells^40,55^. CD4^dim^CD25^hi^Foxp3^+^ cells have previously been shown to have immune suppressive properties and to be reduced in peripheral blood in the second trimester^53^, suggesting accumulation at the fetal-maternal interface^30^. In agreement with these findings, we observed that the proportions and total numbers of CD4^dim^CD25^hi^Foxp3^+^ population decreased from gw 20 to gw 32 and to after delivery, which further strengthens the suggested mechanisms of accumulation of Treg cells at effector sites^25^. An alternative gating strategy, defining CD45RA expression among Treg cells not only excludes false-positive (non-suppressive Foxp3^dim^CD45RA^−^) cells, but also defines the balance between resting (CD45RA^+^Foxp3^dim^) and activated (CD45RA^++^Foxp3^hi^) Treg cells^41^. Similar to the CD4^dim^CD25^hi^Foxp3^+^ population, both aTregs and rTreg decrease in proportion and total number from gw 20 to gw 32 but in contrast the populations are higher after delivery than at gw 32. Consistently, non-pregnant women have higher percentage and number of both populations systemically. As previous shown, subtypes of Tregs have different roles^41^, possibly due to their different chemokine expression^44^.

Differentiated T helper cells may be difficult to investigate in peripheral blood due to the limitation that the lineage specific transcription factors must be intracellularly stained. In an effort to map the peripheral immunity during pregnancy we stained cells for these specific transcription factors, finding that the proportions and numbers of Tbet^+^ memory Th cells were lower in pregnant than non-pregnant women, in line with a previously suggested Th2 deviation during pregnancy^30^. However, in contrast to this suggestion, the numbers (but not proportions) of Th2-associated GATA3^+^ cells were also lower in pregnant women at gw 32. In contrast, the percentage of Th17-associated RORC^+^ cells, with a suggested role as to defend against extracellular bacteria and fungi at mucosal sites, were higher in pregnant women. Other studies suggest that Th17 cells may remain unchanged^58^ or slightly decreased in the periphery during pregnancy^59^ compared to the decidua^60^. The findings on Th subsets in some respects challenge the general view that pregnancy is a Th2 phenomenon, on the other hand this view is increasingly being regarded as over-simplified^30^.

Previous studies have shown increasing numbers of circulating monocytes and granulocytes during pregnancy, and an increased functional activation in these cell subsets^61^, possibly to compensate for the modulated adaptive immunity including a Th2 deviation. In contrast, our results show decreased percentages of CD14^+^ monocytes during pregnancy, although the frequencies of the CD14^+^CD16^+^ subpopulation were increased in pregnant compared to non-pregnant women. This subpopulation, which are in minority compared to the CD14^+^CD16^−^ monocytes, are considered to be more inflammatory^62,63^ and the increased proportions are thus in line with previous findings of increased innate immunity during pregnancy, possibly to compensate for reduced adaptive responses but still maintain fetal tolerance and infection protection.

The number of circulating NK cells was decreased in peripheral blood during pregnancy in our study. Previous studies suggest a type 2 immune deviation also in the NK cell compartment, including reduced IFN-γ secretion^28,29,64,65^. In peripheral blood, NK cells can be divided into major subsets of CD56^dim^CD16^hi^ (~ 90%) and a smaller population of CD56^hi^CD16^−/dim^ (~ 10%)^66^ which are considered to belong to the innate lymphoid cell (ILC) family^67^. These populations differ in several aspects, including cytotoxic potential, cytokine production, and expression of cell surface markers. The CD56^hi^CD16^−/dim^ population is sometimes referred to as regulatory because of its reduced cytotoxic capacity and cytokine producing ability^68^. Interestingly, The CD56^hi^ population was higher during pregnancy, in line with an immune modulatory role. In contrast, the CD56^dim^ population was decreased during pregnancy, explaining the lower frequency of the total NK population, and in line with a lower cytotoxic potential.

The B-cell population, defined as CD19^+^ cells, was increased in pregnant compared to non-pregnant women. This is in line with earlier findings^69^ and possibly related to estrogen production^70^.

In conclusion, some immunomodulatory effects were observed among circulating activated and resting Treg cells after around 20 weeks of treatment with *L. reuteri* during pregnancy, while ω-3 PUFA supplementation had no effect in this substudy. Also, pregnancy was associated with several changes in systemic immune cell populations that indicate tolerance to the fetus while maintaining protection against infections.

## Supplementary information

Supplementary Information 1.

Supplementary Information 2.

Supplementary Information 3.

Supplementary Information 3.

Supplementary Information 5.

Supplementary Information 6.

Supplementary Information 7.

Supplementary Information 8.
