# Supplementary material for: Changes in peripheral immune populations during pregnancy and modulation by probiotics and ω-3 fatty acids
Source: Sci Rep. 2020 Oct 30;10:18723. doi: 10.1038/s41598-020-75312-1 (PMC7599237; doi:10.1038/s41598-020-75312-1)
Supplement: Supplementary file 1 — Supplementary Information 1. [file 41598_2020_75312_MOESM1_ESM.docx]

**Supplementary Figure 1**. Gating strategies for the different cell populations phenotyped in the study. A) T helper cells expressing CD3^+^CD4^+^ was gated from the FSC and SSC, within the CD3^+^CD4^+^ naïve and memory subset was determined (CD45RA^+/-^). Naïve T helper cells CD45RA^+^ was used to determine the gate for differentiated T helper cells in the memory T helper cell population CD45RA^-^ (a maximum of 1 % positive of cells from the naïve population were allowed which were considered as an adjustment for background staining) The gate was transferred to the memory population and used to determine differentiated T helper cells expressing lineage transcription factors, in the method optimization an isotype was used to validate the method and intracellular staining, but not used in the actual study B) CD45RA^+^Tbet^+^/GATA3^+^ and C) CD45RA^-^ Tbet^+^/GATA3^+^ D) CD45RA^+^ RORC and E) CD45RA^-^RORC^+^. Foxp3 was not used in this gating strategy since it is known that activated T cells without suppressing capacity can express Foxp3. Therefore, established gating strategies for Treg cells were used. Treg subtype gating F) (CD3^+^CD4^+^), CD4^dim^CD25^hi^Foxp3^+^ G) intracellular FoxP3 expression in CD4^dim^CD25^hi^Foxp3^+^ and CD3+CD25- cells to prove expression of Foxp3 in Treg cells, H) CD4^+^CD45RA^+/-^Foxp3^+/++^ as defined in ^41^ I) Intracellular FoxP3 expression in CD4^+^CD45RA^+/-^Foxp3^+/++^  as defined in ^41^ .Lymphocytes in whole blood based on FSC/SSC J) NK cells (CD56^dim^/^hi^) and K) B cells (CD19^+^) L) CD4^+^ and CD8^+^ cells. M) Monocytes expressing CD14^+^CD16^+/-^

**Supplementary Figure 2.** Lymphocyte populations during pregnancy and in non-pregnant women A) percentage of lymphocytes B) number of lymphocytes C) percentage of CD4^+^ cells D) number of CD4^+^cells E) percentage of CD8^+^ cells F) number of CD8^+^ cells G) percentage of CD19^+^ cells H) number of CD19^+^ cells I) percentage of CD56^+^ cells J) number of CD56^+^ cells K) percentage of CD56^dim^ cells L) number of CD56^dim^ cells M) percentage of CD56^hi^ cells N) number of CD56^hi^ cells. Median values and interquartile ranges are shown. Mann-Whitney *U*-test and Wilcoxon test were used for statistical comparisons.

**Supplementary Figure 3.** Naïve and memory cells during pregnancy and in non-pregnant women A) percentage of (CD4^+^CD45RA^-^) cells B) number of (CD4^+^CD45RA^-^) cells C) percentage of (CD4^+^CD45RA^+^) cells D) number of (CD4^+^CD45RA^+^) cells. Median values and interquartile ranges are shown. Mann-Whitney *U*-test and Wilcoxon test were used for statistical comparisons.

**Supplementary Figure 4.** Monocytes during pregnancy and in non-pregnant women A) percentage of CD14^+^ monocytes B) percentage of CD14^+^CD16^-^ cells among CD14^+^ monocytes C) percentage of CD14^+^CD16^+^ cells among CD14^+^ monocytes. Median values and interquartile ranges are shown. Mann-Whitney *U*-test and Wilcoxon test were used for statistical comparisons.
