# Supplementary figures and images for: Changes in peripheral immune populations during pregnancy and modulation by probiotics and ω-3 fatty acids
Source: Sci Rep. 2020 Oct 30;10:18723. doi: 10.1038/s41598-020-75312-1 (PMC7599237; doi:10.1038/s41598-020-75312-1)

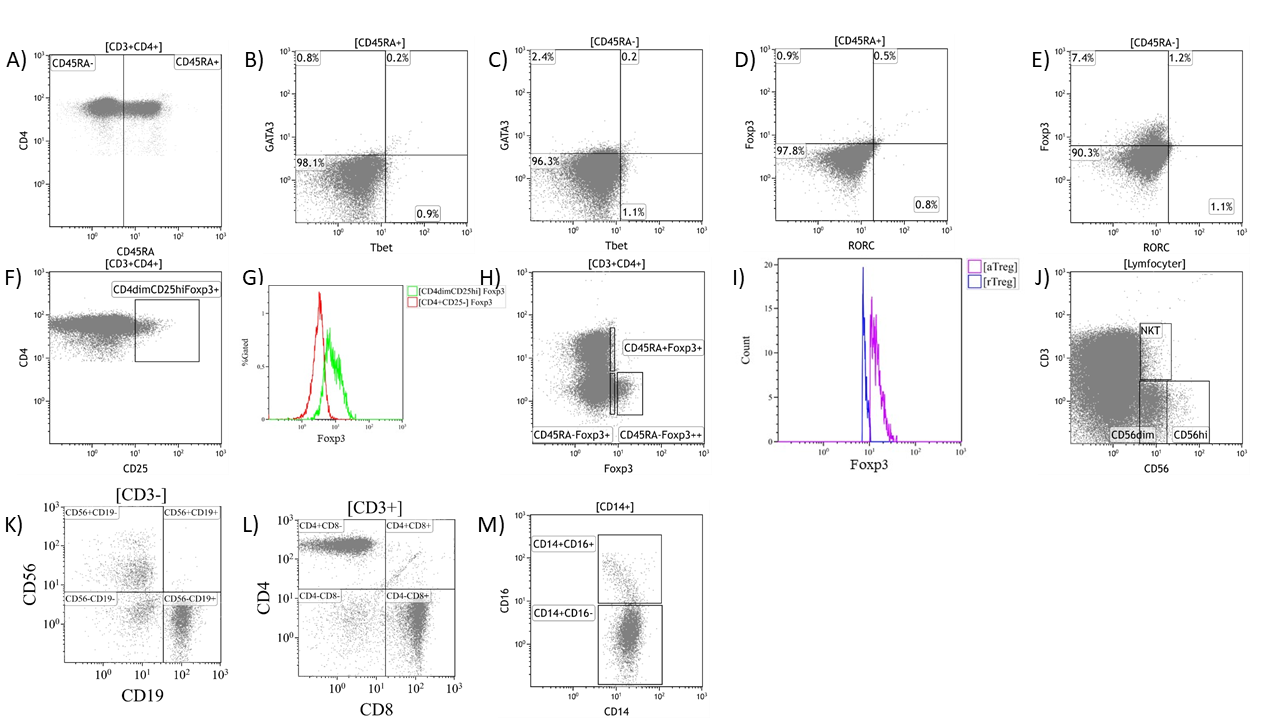

Supplement: Supplementary file 2 — Supplementary Information 2. [file 41598_2020_75312_MOESM2_ESM.png]

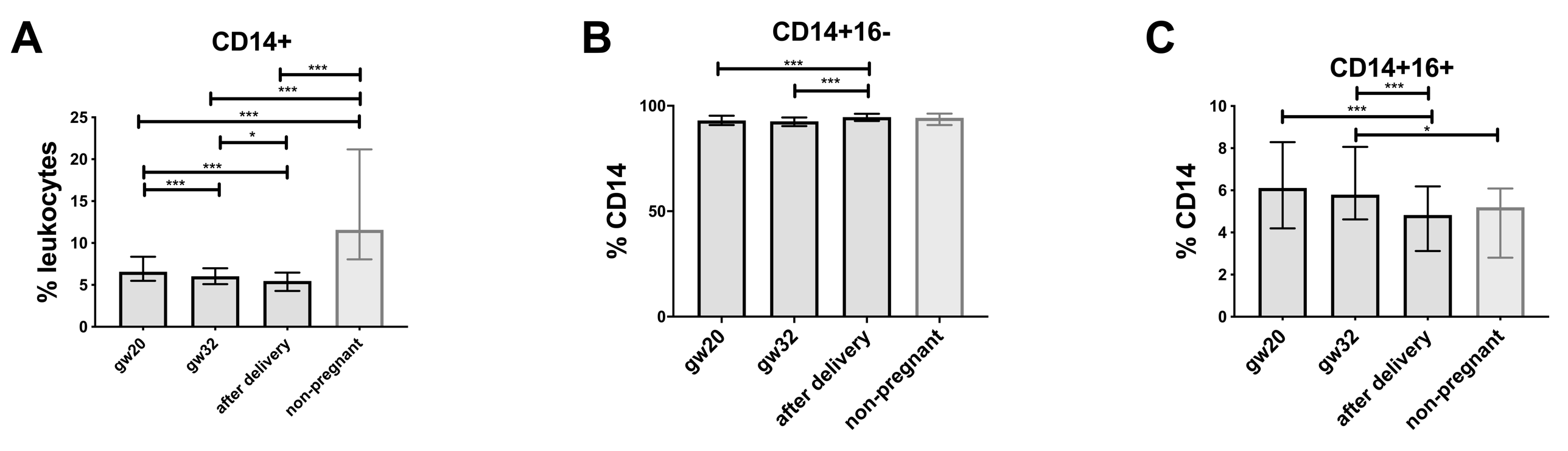

Supplement: Supplementary file 5 — Supplementary Information 5. [file 41598_2020_75312_MOESM5_ESM.jpg]
