# Supplementary material for: Changes in peripheral immune populations during pregnancy and modulation by probiotics and ω-3 fatty acids
Source: Sci Rep. 2020 Oct 30;10:18723. doi: 10.1038/s41598-020-75312-1 (PMC7599237; doi:10.1038/s41598-020-75312-1)
Supplement: Supplementary file 6 — Supplementary Information 6. [file 41598_2020_75312_MOESM6_ESM.docx]

| **Supplementary Table I**. **Clinical descriptive of participating non-pregnant women** | |
| --- | --- |
| Age at inclusion (mean) | 29.1 |
| Use of hormonal contraceptives (yes /no) | 0/22 |
| Menstrual cycle (luteal/follicular)^a^ | 9/11 |
| Smoking (yes/no) | 0/22 |
| Previous births (yes/no) | 6/16 |
| Any allergic disease, ARC (yes/no) | 3/19 |

^a^based on a 28-day menstrual cycle, data not available for 2 individuals
