# Supplementary material for: Changes in peripheral immune populations during pregnancy and modulation by probiotics and ω-3 fatty acids
Source: Sci Rep. 2020 Oct 30;10:18723. doi: 10.1038/s41598-020-75312-1 (PMC7599237; doi:10.1038/s41598-020-75312-1)
Supplement: Supplementary file 7 — Supplementary Information 7. [file 41598_2020_75312_MOESM7_ESM.docx]

**Supplementary Table II. Antibodies used for flowcytometry phenotyping.**

| **Antigen** | **Flourochrome** | **Antibody** | **Clone** | **Supplier** |
| --- | --- | --- | --- | --- |
| CD3 | APC-H7 | Mouse, IgG1, κ | SK7 | BD |
| CD4 | PE-Cy7 | Mouse, IgG1, κ | SK3 | BD |
| CD8 | FITC | Mouse, IgG1, κ | SK1 | BD |
| CD14 | FITC | Mouse, IgG1, κ | M0P9 | BD |
| CD16 | PerCP-Cy5.5 | Mouse, IgG1, κ | 3G8 | BD |
| CD19 | APC | Mouse, IgG1, κ | SJ25C1 | BD |
| CD25 | PerCpCy5.5 | Mouse, IgG1, κ | M-A251 | BD |
| CD45RA | HV450 | Mouse, IgG2b, κ | HI100 | BD |
| CD56 | PerCP-Cy5.5 | Mouse, IgG1, κ | B159 | BD |
| FoxP3 | FITC | Rat, IgG2a | PCH101 | eBiosciences |
| GATA3 | PE | Rat, IgG2b, κ | TWAJ | eBiosciences |
| isotype | PE | Rat, IgG2b, κ | eB149/10H5 | eBiosciences |
| isotype | eFlour660 | Mouse, IgG1, κ | P3.6.2.8.1 | eBiosciences |
| RORC | PE | Rat, IgG2a | AFKJS-9 | eBiosciences |
| T-bet | eFlour660 | Mouse, IgG1, κ | eBio4B10 | eBiosciences |
