# Supplementary material for: Changes in peripheral immune populations during pregnancy and modulation by probiotics and ω-3 fatty acids
Source: Sci Rep. 2020 Oct 30;10:18723. doi: 10.1038/s41598-020-75312-1 (PMC7599237; doi:10.1038/s41598-020-75312-1)
Supplement: Supplementary file 8 — Supplementary Information 8. [file 41598_2020_75312_MOESM8_ESM.docx]

**Title page for supplementary files**

Changes in peripheral immune populations during pregnancy and modulation by probiotics and ω-3 fatty acids

Forsberg A^1^, Abrahamsson TR^2^, Nilsson L^3^, Ernerudh J^4^, Duchén K^2^, Jenmalm MC^1^

Supplementary Figure 1

Supplementary Figure 2

Supplementary Figure 3

Supplementary Figure 4

Supplementary Table 1

Supplementary Table 2
